# A Look into Pharmacy Practices among the Purépecha Indigenous Community

**DOI:** 10.3390/pharmacy11040118

**Published:** 2023-07-19

**Authors:** Katrin Henry, David R. Axon

**Affiliations:** 1 Department of Pharmacy Practice & Science, R. Ken Coit College of Pharmacy, The University of Arizona, 1295 N. Martin Ave., Tucson, AZ 85721, USA; 2 Center for Health Outcomes and PharmacoEconomic Research (HOPE Center), R. Ken Coit College of Pharmacy, The University of Arizona, 1295 N. Martin Ave., Tucson, AZ 85721, USA

**Keywords:** Purépecha, indigenous, pharmacy, pharmacy practice, traditional environmental knowledge, traditional medical knowledge, primary health care

## Abstract

This report describes the adoption and integration of Western medicine in Cherán K’eri after the social changes in the 1940s which led to the transition from healer to pharmacist. There are various health models that rely heavily on community pharmacies. The place used as the basis for this report was a clinic managed by a Purépecha-speaking physician and pharmacist that served primarily monolingual indigenous Purépecha patients, whose population was around 9550 according to the 2010 census. Twelve major differences were observed between community pharmacies in the United States and the community pharmacies of Cherán. It was also observed that the modern approach to the health of the indigenous population used a combination of Western medicine together with traditional methods and only resorted to short-term therapies with Western medicines lasting five days or less. A formulary from the clinic’s community pharmacy compiled in 2022 listed the 38 most common medications. Medications used included anti-infectives (*n* = 3), central nervous system (*n* = 2), endocrine/hormonal (*n* = 3), gastrointestinal (*n* = 3), musculoskeletal (*n* = 17), respiratory or allergy (*n* = 6), and genitourinary (*n* = 2).

## 1. Introduction

Pharmacists, and other healthcare professionals, provide healthcare services daily to millions of adults who use the United States (US) healthcare system each day. Healthcare professionals are typically trained in and practice Western medicine, which meets the needs of most of their patients. Few professionals have a thorough understanding of the approach to health of other populations. North America is home to many indigenous peoples, including the Purépecha. In January 2022, a student pharmacist from the University of Arizona R. Ken Coit College of Pharmacy, who is also a Purépecha tribal member, visited Cherán K’eri and volunteered at a Purépecha clinic. The following report contains a synthesis of her observations from an immersion experience that may help raise awareness of alternative pharmacy practice systems.

The Purépecha, varied in spelling such as Purepeche, P’urépecha, P’urhépecha, Phurépecha, P’orhépecha, or Phorhépecha, live in the mountainous pine tree forests of the state of Michoacán, Mexico, at elevations reaching up to 10,000 feet. The Purépecha formed a centralized empire as early as 1350 [1]. The Purépecha empire rivaled and was never conquered by the Aztec empire. The encounter with the Spanish occurred after the fall of the Aztec capital in 1521. Records show the people had been known to the Aztecs as the “Michoacan” or “place of fishermen” but were referred to as “Tarascans” or “father-in-law” by the Spanish. When asked about their origin, the Purépecha described themselves to the Spanish as the recent arrivals from the backs of turtles in a journey by sea [2].

The Purépecha were isolated until the paving of an interstate highway between Guadalajara and Mexico City in the 1940s. With a new inroad into the society, academic investigations delved into the Purépecha way of life, but little material was produced about their approach to health. Attempts to research medicinal knowledge is challenging in indigenous communities [3].

The Purépecha tribal identity was legally recognized in Mexico after the *levantamiento* (uprising) in 2011 and reaffirmed by the United Nations in 2013 [4]. In Cherán K’eri, tribal customs and uses established over 500 years ago continue to govern the people today. The core of Purépecha speakers live within twenty-one municipalities in the sierras of the State of Michoacán, Mexico [5]. The size of the tribe is estimated to be 154,943 persons based on the number of indigenous speakers in Michoacán [6].

Prior to the opening of the first pharmacy, stores sold general merchandise that included only a few pharmaceutical products. There were no pharmacists and only one unlicensed medical doctor. The curers provided most of the healthcare from products they brought from larger cities or medicinal herbs. The curers were different from physicians, midwives, chemists, curanderos (traditional native healer), witches, etc. Some of the plants that grow in the territory of the Purépecha have unique medicinal properties. One example of a medicinal herb is the woody shrub from the mountains, cenicilla. Water is boiled and half a handful of dried cenicilla leaves and stems are added. After a few minutes of seeping, the very bitter tea is strained into a mug and ready to drink. One mug is enough to stop gastrointestinal issues. The stems and leaves are dried again to be used a second time. The older women who forage in the mountains know how to choose the best cenicilla.

## 2. To Live in Cherán K’eri Is to Live Close to Nature

Throughout the immersion experience in mid-January 2022, the temperature was about 4 °C at night and 18 °C during the day. At an elevation of almost 10,000 feet, the temperature was expected to be consistently cold throughout the year. Fire was used for cooking and heating throughout the entire day, evening, and part of the night. Over time, the soot blackened the rafters and made a nice color contrast to the blue sky above that was seen through the smoke escape. It was said that the wind during the dry season from May to August carried up the fertile volcanic dust into the air. Interestingly, breathing-related health issues were rare and not a single inhaler was sold at the pharmacy. Rather, the soot and dust seemed to contribute to earwax buildup. Rainwater was collected in a tank that was placed on top of the roof during the rainy season; it was also sourced from community wells and stored in containers. Sometimes the water was boiled, other times people drank it as it came out from the rocks. Electricity was not used very often, not even to heat water. The locals said cold showers were better for the body than hot showers. People used to bathe in the river. Rooms were also not heated because the temperature change led to body aches. The kitchen and bedroom furniture were made from the good pine tree wood and decorated with elaborate carvings. Volcanic rock was used as the building material for walls, chimneys, and fire pits. Clothing was handmade. A complete outfit took one year to elaborately embroider with brightly colored patterns, plants, and animals, and was worn in everyday work. Bells from a church belltower sounded a few times in the day, but time was mostly forgotten.

## 3. Various Pharmacy Models Exist within the Health System of the Purépecha

Even by the 1940s, no pharmacy existed in Cherán. Mild ailments were treated at home. The cultivation of medicinal herbs at home was one common source of remedy. Serious sickness was treated by šuríjki (curers), women of mature age whose knowledge was passed down generationally, as far back as their grandmothers. Treatment often involved products from pharmacies outside Cherán. Curers focused their expertise on one to upwards of three illnesses. Treatment could be obtained at the curer’s house, or a curer would visit a patient’s house for no extra charge. A physician could be called to come from a clinic in Parácho, a neighboring town, but doing so was expensive. Curers were among other professions improving community health like midwives, physicians, and others not mentioned in this report. At that time, ideas of sickness and cure were in a state of transition from traditional medicine to Western medicine [7].

The first pharmacy opened in 1961 after a traveler from Uruapan decided to sell traveler’s medicine. By 2022, many shops were independent pharmacies, and some collaborated with physicians. The student counted at least twelve pharmacies during a ride through the town. One pharmacy was known for its expertise in children’s medicines and a pediatrician was on-site. Another pharmacy collaborated with a physician to provide services for the monolingual patients in Purépecha. Apart from pharmacies and physicians’ offices, one local community hospital and one newly opened dialysis clinic served the community. A curer’s profession had transformed into that of a pharmacist.

The Community Government Greater Council of the Purépecha does not regulate medical or professional licenses, as is the case in the US. Persons can establish a pharmacy, clinic, etc., if they are tribal members and have the skill sets to do so from formal training or from a family trade. As the Purépecha tribe adopted Western medicine and Western ways, families sent their children to attend professional schools in engineering, business, and medicine, etc. Thus, the younger generation are typically licensed to practice the certain skill set they choose for their profession, while the older generations may not be, having simply followed the family's trade and forgone any formal training. 

The physician whose clinic was observed for this report completed formal medical training outside of Cherán but returned to practice medicine in Cherán. The pharmacist observed in this report completed formal training in dentistry outside of Cherán, and co-manages a dental studio separate from the clinic. Rather than having formal pharmacy training, this pharmacist practices pharmacy as a family trade, where the knowledge has been passed down from the family’s šuríjki (curer) over several generations. The pharmacist joined together with the physician's office to expand the services they offer as a clinic. 

## 4. The Clinic for Indigenous Patients in Their Language

The clinic used as a basis for this report was unique as indigenous patients had access to both medical and pharmacy care in the indigenous language. Both the physician and pharmacist provided services in the native language. To give some perspective on the patient base, 9550 (1.1%) persons in Mexico were monolingual Purépecha (Tarasco) language speakers as per the 2010 census [8]. At the clinic there was a physician’s office, a room with patient beds, and a pharmacy. The pharmacy was open from 10A.M. to 2P.M. and again from 4PM to 8PM every day except Sunday. The physician’s hours were the same as the pharmacy and remained flexible to accommodate community visits during non-busy days or after-hour care. The physician would regularly walk into the pharmacy to check the medications on-hand and to discuss patient care plans with the pharmacist.

During the student’s immersion experience, she had an opportunity to assist the doctor with his daily appointments. A common visit to the physician was for the removal of impacted ear wax. The student assisted in this procedure. The physician combined boiling hot water and the ambient temperature cold water to make lukewarm water. Then, the physician used a syringe to stream water into the patient’s ear canal. A water basin was held under the ear and the head steadied slightly downward. The physician frequently asked the patient how the temperature of the water felt and how well she could hear. After one hour, the plugs were partially removed. The physician and the patient had a lively conversation in Tarasco, as was typical with each patient during their visit. “The breads at their wedding!” she indicated the size with her arms in a circle. At the end of the procedure, the physician instructed the patient to use an oil to soften the wax prior to coming to the consultorio (physician’s office) the following week to complete the procedure.

## 5. The Adoption of Western Medicine Evidenced in Modern Pharmacy Practices

The student pharmacist observed and documented that the clinic’s pharmacy was managed by the pharmacist and, at times, the immediate family assisted customers at the counter. Medications were sourced from Mexico and instructions were printed only in Spanish. The pharmacist provided medication counseling in Purépecha. Two generations ago, the pharmacy switched from selling traditional medicine to brand-name medications. The pharmacy sold brand-name products instead of generic products per consumer choice. One antibiotic, clindamycin, was available in two dosage forms. One dosage form was a generic clindamycin 300 mg capsule and the other was brand-name clindamycin 75 mg/5 mL granules for oral solution. In this pharmacy, there were 38 most commonly used medications, known within the retail pharmacies of the US as “fast movers”. The pharmacist confirmed most of the medications sold were for the common cold and for pain. Most medications at this pharmacy required a prescription. Table 1 displays a formulary of the most used drugs at this pharmacy compiled by the student during the visit. Translations to the US pharmacopeia were completed using IBM Micromedex [9], DailyMed [10], and PubMed.

The student also observed many differences in pharmacy practice at this site serving an indigenous population compared to a community pharmacy in the US. A few of the differences are summarized in Table 2.

## 6. Integration of Western Medicine Accepted for Short-Term Duration of Therapy

At-home traditional teas or foods to treat illness were still common. Medicinal plant and animal knowledge was shared within families. The dried leaves and stems of medicinal herbs were sold at the open-air market by women who dedicated themselves to finding them in the mountains. A trip to the physician’s office or pharmacy was made only after the person could not manage the pain or other conditions themselves. Many indigenous patients forwent medication entirely because of feeling worse from side effects and perceptions of possible life-threatening interactions. Long-term medication therapy for chronic diseases was not widely accepted, so a typical sale at the pharmacy was a few pills of a couple different medications.

## 7. The Effect of the COVID-19 Pandemic on Cherán K’eri

The population of Cherán K’eri was 20,586 in 2020 [6]. By January 2022, about 400 (1.9%) had died based on observations by the townspeople. Cherán K’eri did not participate in testing nor in counting cases.

## 8. Conclusions

This report has documented how the Purépecha have adopted Western medicine practices since the 1940’s when they were still an uncontacted tribe. The transition on the ideas of sickness among the Purépecha paralleled the redefinition of health practitioners, most notably the change from curer to pharmacist. In addition, the Purépecha physician–pharmacist team provide health services for monolingual Purépecha patients. The widespread adoption and integration of Western medicine was led by pharmacies. Post-1961, medications for pain and symptoms of the common cold were the most common among one indigenous clinic of the Purépecha. A list of these medications was organized into a formulary as a highlight of this report on pharmacy practice. The list of 39 medications leads to further questions. Formularies vary across different health systems depending on the health needs of the population. How does this formulary compare to the formularies of other health systems? What are the safety benefits associated with a smaller number of medications? A future study may be able to explore whether an intervention for a smaller number of medications improves patient health.

## Figures and Tables

**Table 1:** Formulary for an indigenous community pharmacy of Cherán K’eri.

Drugs: Generics, BRAND	
Cherán K’eri	United States
Anti-Infectives (*n* = 5)	
amoxicilina, AMOXIL	amoxicillin
amoxicilina/ác clavulánico, CAXICUM	amoxicillin/clavulanate potassium, AUGMENTIN
dicloxacilina, AMIFARIN	dicloxacillin
clindamicina	clindamycin, CLEOCIN
clindamicina, DALACIN C	clindamycin, DALICIN C
Central Nervous System (*n* = 2)	
dimenhidrinato, DRAMAMINE	diphenhydramine, DRAMAMINE (OTC)
ondansetron, ZOFRAN	ondansetron, ZOFRAN; ZUPLENZ
Endocrine/Hormone (*n* = 3)	
dexametasona, BEADVANCE	dexamethasone, DECADRON; DXEVO 11-DAY; HEMADY; HIDEX 6-DAY; TAPERDEX 12-DAY; TAPERDEX 6-DAY; TAPERDEX 7-Day; ZCORT 7-DAY
parametasona, DILAR	paramethasone
parametasona/clorfenamina DILARMINE	paramethasone/chlorphenamine
Gastrointestinal (*n* = 3)	
bicarbonato potásico/cloruro de potasio/lisina, CORPOTASIN CL	potassium bicarbonate/potassium chloride/lysine
hidróxido de aluminio/óxido de magnesio/simeticona, ESPAVÉN ALCALINO	aluminum hydroxide/magnesium oxide/simethicone
neomicina/coalín/pectina, TREDA	neomycin/kaolin/pectin
Musculoskeletal (*n* = 17)	
ácido acetilsalicílico, ASPIRINA	acetylsalicylic acid, ASPIRIN (OTC)
ácido acetilsalicílico/cafeína, CAFIASPIRINA	acetylsalicylic acid/caffeine
ácido acetilsalicílico/fenacetina/acetanilida/ cafeína, NORDINET ADULTO	acetylsalicylic acid/phenacetin/acetanilide/caffeine
benzocaína, GRANEODÍN B	benzocaine
clonixinato de lisina, DORIXINA	lysine clonixinate
diclofenaco/carisoprodol, DOLAREN	diclofenac/carisoprodol
diclofenaco/vitaminas complejo B, DOLO-NEUROBIÓN	diclofenac/vitamin B complex
flurbiprofeno, GRANEODÍN F	flurbiprofen
ketorolaco, DOLAC	ketorolac, DOLAC
ketorolaco/tramadol, MAVIDOL TR	ketorolac/tramadol
metamizol magnésico, MAGNOPYROL	dipyrone (metamizole)
metamizol sódico, NEO-MELUBRINA	dipyrone (metamizole)
naproxeno sódico, ALLIVAX	naproxen, ANAPROX DS
paracetamol/ácido acetilsalicílico/cafeína, MIGRAÑA	acetaminophen/acetylsalicylic acid/caffeine, BAYER MIGRAINE FORMULA (OTC)
paracetamol/clorhidrato de fenilefrina/bromhidrato de dextrometorfano/succinato de doxilamina, TABCIN NOCHE	acetaminophen/phenylephrine/dextromethorphan/doxylamine
paracetamol, MEJORALITO PEDIÁTRICO	acetaminophen
paracetamol/cafeína, SEDALMERCK MAX	acetaminophen/caffeine
Respiratory, Allergy (*n* = 6)	
ácido acetilsalicílico/fenilefrina/clorfenamina, TABCIN 500	acetylsalicylic acid/phenylephrine/chlorpheniramine
clorfenamina/fenilefrina/paracetamol, DESENFRIOL D	chlorpheniramine maleate/phenylephrine HCl/acetaminophen, CONTAC COLD + FLU NIGHT FORMULA (OTC)
clorfenamina/fenilefrina/paracetamol, DESENFRIOL-ITO PLUS	chlorpheniramine/ phenylephrine/acetaminophen
paracetamol/clorhidrato de fenilefrina, THERAFLU TD	acetaminophen/ phenylephrine HCl
paracetamol/clorhidrato de fenilefrina/bromhidrato de dextrometorfano/maleato de clorfenamina, TABCIN ACTIVE	acetaminophen/phenylephrine HCl/dextromethorphan/chlorpheniramine maleate
paracetamol/clorhidrato de fenilefrina/maleato de clorfenamina, CONTAC ULTRA; THERAFLU THERAPILLS	acetaminophen/phenylephrine HCl/chlorpheniramine maleate, CONTAC COLD + FLU NIGHTTIME
Genitourinary (*n* = 2)	
piperidolato, DACTIL OB	-
sildenafil, EDEGRA	sildenafil, VIAGRA

**Table 2:** Observed differences in Purépecha compared to Western pharmacy practices.

	Description of Differences
1.	Only one medication was available for hypertension, losartan, and it was not commonly sold.
2.	No insulin was sold even though patients were diabetic. Metformin was the only medication for pre-diabetes and diabetes, available in two strengths of 500 mg and 1000 mg, and was not commonly sold.
3.	No medication was available for hypercholesteremia.
4.	No medication was available for cardiovascular disease.
5.	No dermatologic medications were available except for topical penicillin ointments.
6.	Only one ophthalmic medication was available, Bausch + Lomb Ocuvite, and no eyedrops.
7.	No inhalers were available. Breathing problems were rare and treated with teas.
8.	Beside pain medications, the next most common medications were anti-inflammatories. One opioid medication was dispensed, tramadol, in combination with ketorolac. Many medications were injectable. Patients took the ampules home with needles and syringes and injected themselves.
9.	Most medications were prescribed for a therapy lasting five days or less.
10.	Prices were negotiable. Insurance did not exist.
11.	All brand-name and generic medications were from Mexico.
12.	All products were behind the counter including self-care products and antacids.

## Data Availability

Not applicable.
